# Supplementary material for: merlin, an improved framework for the reconstruction of high-quality genome-scale metabolic models
Source: Nucleic Acids Res. 2022 Jun 13;50(11):6052–66. doi: 10.1093/nar/gkac459 (PMC9226533; doi:10.1093/nar/gkac459)
Supplement: gkac459_Supplemental_Files [file gkac459_supplemental_files.zip › S1.pdf]

merlin v4.0: an updated platform for the reconstruction of high-quality genome-scale metabolic models

João Capela[1,2], Davide Lagoa[1,2], Ruben Rodrigues[1,2], Emanuel Cunha[1,2], Fernando Cruz[1,2] Ana Barbosa[1,2], José Bastos[1,2], Diogo Lima[1,2], Eugénio C. Ferreira[1,2], Miguel Rocha[1,2], Oscar Dias[1,2,*]

[1] Centre of Biological Engineering, University of Minho, 4710-057, Braga, Portugal

[2] LABBELS – Associate Laboratory, Braga/Guimarães, Portugal

* To whom correspondence should be addressed.

Tel: 00351 253 604 422

Email: odias@deb.uminho.pt

Supplementary data 1 – *merlin's* plugins implementation, databases and models

ORM

The new features available in the newest version require the storage and manipulation of complex biological data. In the previous version, the interaction between the MySQL (https://www.mysql.com/) database and the biological data involved prepared statements. Database maintenance and management using prepared statements can be a time-consuming task that requires technical skills and may hinder the development of new features.

To address this problem, two frameworks, Hibernate (https://hibernate.org/) and Spring Data (https://spring.io/projects/spring-data) were integrated into *merlin*'s engine. Hibernate is an open-source ORM framework created to simplify development, allowing the conversion of Plain Old Java Objects (POJO) classes into database tables by using mapping configurations. The data present in a Java object is automatically converted into Structured Query Language (SQL) data without the development of SQL queries, required for the prepared statements. The adoption of an ORM framework improved the database maintenance, as any modification of Java entities can be automatically reflected into the database schema, facilitating the development of database migration patches between versions of *merlin* and its plugins. Another improvement prompted by the ORM adoption was the option to use the H2 database (http://h2database.com/), a Java embedded database, which overrides the technical requirement of installing MySQL in the machine that will run *merlin*.

Spring data was integrated into *merlin's* engine to foster Hibernate framework functionalities and further improve *merlin*'s database management. In the new version of *merlin*, Spring-based data accesses manage the database operations performed by Hibernate ORM and *merlin* CORE data

structures. The data accesses split the database operations from *merlin*'s operations, enabling the development of improved data storage schemas, transparent to *merlin*'s operations, which allows integrating different types of database, e.g. non-relational databases like MongoDB (https://www.mongodb.com/) or Neo4J (https://neo4j.com/).

Table S1 – Databases used by *merlin.*

Database / Online resource	Description	Operations using the database	Access	Reference
Kyoto Encyclopedia of Genes and Genomes (KEGG)	KEGG is an integrated resource encompassing 16 different databases divided by categories. These categories are divided into Systems, Genomic, Chemical, and Health information. The Systems category comprises pathways, modules, as well as relevant hierarchies of different biological entities. The Genomic category includes genes, genomes, as well as valuable genome annotations. The Chemical one compiles metabolic and biochemical information relevant for building up a metabolic network. The Health category includes drugs and health related substances.	• Importing metabolic data;<br>• Performing GPR associations;<br>• "Draw in Browser" operation.	Rest API through http://rest.kegg.jp/	(1)
National Center for Biotechnology Information (NCBI)	NCBI comprises databases with vast application in biomedicine and biotechnology, including bioinformatics tools with free usage. It houses a database for multiple genomes from all domains of life, as well as a taxonomic hierarchy regularly updated, providing relevant information in the context of metabolic modelling.	• Importing genomes<br>• Retrieving taxonomy lineage	Using Entrez taxonomy and Entrez Proteins databases through https://eutils.ncbi.nlm.nih.gov/entrez/eutils	(2)
ModelSEED	ModelSEED is an integrated database that includes information from different databases and GSM models. The biochemical data therein present involves reactions' and metabolites' compartmentalization, transport reactions, charged molecules and proton balancing. Moreover, annotations and database's cross-references are provided.	• Importing metabolic data<br>• Biomass Templates<br>• Cross-references for metabolites and reactions	Files download from https://github.com/ModelSEED/ModelSEEDDatabase	(3)
Transporter Classification Database (TCDB)	TCDB is a freely available resource that accounts for the enumeration and curated characterization of transporter systems from varied types of living organisms. It includes a classification system for transporters that can incorporate functional and phylogenetic information. This system is approved and adopted by the International Union of Biochemistry and Molecular Biology (IUBMB).	• Using TranSyT for transporter systems annotation	Using TranSyT's auto-updatable TCDB's database	(4)
Universal Protein Resource Knowledgebase (UniProtKB)	UniProtKB is a knowledgebase of functional information on proteins. It includes two main databases: Swiss-Prot and TrEMBL. The former corresponds to manually curated information, whereas the later contains unreviewed and automatically generated data.	• Reference database to perform the enzymes annotation	Through EBI's remote access	(5)

Gastric Cancer Platform	GCPlatform is a novel knowledge-base and web portal to foster scientific discovery and drug development efforts in gastric cancer research, developing novel methods to integrate data from different origins, results of data analysis, and data mining tools. It houses protein information from multiple sources (e.g. Uniprot, ProteomicsDB, InterPro) and provides summarized protein information with repository cross-referencing.	• Identifier mapping and enzyme information retrieval after performing alignments	Rest API through https://gcplatform.bio.di.uminho.pt/api/prot/find/ids	-

Table S2 – Merlin's plugins' implementation and references.

Reconstruction step	Tool	Implementation	Third-party software	Reference
Genome annotation	*SamPler**	Integrated in *merlin*	no	(6)
	Automatic workflow*	Integrated in *merlin*	no	-
	*TranSyT**	Remote access to *TranSyT*'s RestAPI and parsers to integrate the results;	no	(7)
	Comparmentalization*	Web-scrapers for *LocTree3* (https://rostlab.org/services/loctree3/) and *WolfPSort* (https://wolfpsort.hgc.jp/) reports' webpage, and report parser for *PSort3b* (https://www.psort.org/psortb/)	yes	(8-10)
Model reconstruction	GPR associations	Integrated in *merlin*, using KEGG API	no	(11)
	Biomass formulation*	Integrated in *merlin*	no	(12)
	Draft Reconstruction*	Integrated in *merlin*	no	-
	Draft Reconstruction*	BiGG Integration Tool - Access to an internal RestAPI – running in BioSystems servers	no	(13)
Curation	*BioISO**	Remote access to *BioISO*'s RestAPI and parsers to render the results	no	(14)
	*MEMOTE**	Access to an internal RestAPI – running in BioSystems servers – and parser to render the results	yes	(15)
	*EscherMaps**	Access to an internal RestAPI – running in BioSystems servers – opening results HTML in user's default browser	yes	(16)
	*MetExploreViz**	Web-service run locally - remote access to *MetExploreViz*'s RestAPI	yes	(17)

* New features of version 4.0

Table S3 - Published models reconstructed in merlin.

Type	Organism	Applications	Metabolites	Reactions	Genes	Compartments	Remarks	Reference
Gram-negative bacteria	*Helicobacter pylori 26695*	Pathogen	412	640	383	3	191 new metabolic functions were identified in the genome re-annotation. Better phenotypical predictions were obtained in the specific growth rate comparing to other models reconstructed with other tools. Agreement with experiments using glutamate as sole carbon source, whereas its analogous were not capable of predicting growth under glutamate consumption. As for the gene essentiality, it performed slightly better than its analogous, using experimental data to corroborate it.	(18)
	Streptococcus pneumoniae R6	Pathogen	489	596	372	2	First model for any *S. pneumoniae* strain. Complete functional annotation of the genome: 372 metabolic genes identified out of the 2046 candidates. Accurate phenotypical predictions under different environmental conditions, different carbon sources and oxygen availability, being corroborated by 5 different studies. 67 essential genes not listed in OGEE database were identified.	(19)
	Actinobacillus succinogenes 130Z	Bioproduction of succinic acid	713	1072	722	-	Provided a better comprehension of *A. succinogenes* metabolism and capacities. Naturally, this organism is one of the major producers of succinic acid and the reconstructed model presents advantages over other SA-producing strains, such as its ability to consume low-cost carbon sources and C5, C6 sugars.	(20)
Gram-positive bacteria	*Streptococcus thermophilus LMD-9*	Food biotechnology	794	962	431	2	Allowed the comparison of their fermentative behaviour in different environmental conditions with other Lactic Acid Bacteria.	(21)
	Lactobacillus acidophilus La-14	Food biotechnology	574	677	470	2		
	Lactobacillus helveticus CNRZ32	Food biotechnology	562	618	389	2		
	Lactobacillus rhamnosus GG	Food biotechnology	740	876	651	2		
	Enterococcus faecalis V583	Pathogen	576	603	366	2	First model of this species. Phenotypical predictions were in accordance with results from chemostat experiments, being able to accurately predict all the organism's end-products comparing to the distribution of fluxes observed in vivo.	(22)

Continued on the next page.

Table S3 – Continued from the previous page.

Type	Organism	Applications	Metabolites	Reactions	Genes	Compartments	Remarks	Reference
Fungi	*Kluyveromyces lactis*	Food biotechnology	1476	1867	906	4	First model of this species: 45 genes updated from the previous genome annotation, 22 new metabolic genes were identified. In silico growth predictions in agreement with Biolog experiments and the online catalogue of strains CBS-KNAW. Accurate predictions using 9 different carbon sources. Replication of 90% of the knockouts from several experiments published over the last three decades.	(23)
	Candida albicans	Pathogen	926	1221	781	4	First model of this species. It correctly predicted 78% of the identified essential genes (84 out of 108 validated experimentally). Proved to be accurate prediction of anaerobic growth utilizing different carbon and nitrogen sources.	(24)
Plantae	*Quercus suber*	Cork production	6481	6230	7871	8	First model of a ligneous tree. Besides the generic model, were also obtained tissue-specific models, for leaf, inner bark, and phellogen, and a diel multi-tissue model.	(25)

Table S4 – *merlin's* research impact as of 2015.

Authors	year	Title	Journal/Book/Conference
**Machado,D., Herrgård,M.J. and Rocha,I.**	2016	Stoichiometric Representation of Gene–Protein–Reaction Associations Leverages Constraint-Based Analysis from Reaction to Gene-Level Phenotype Prediction	PLOS Computational Biology
**Jouhten,P., Boruta,T., Andrejev,S., Pereira,F., Rocha,I. and Patil,K.R.**	2016	Yeast metabolic chassis designs for diverse biotechnological products	Scientific Reports
**Pfau,T., Christian,N., Masakapalli,S.K., Sweetlove,L.J., Poolman,M.G. and Ebenhöh,O.**	2016	The intertwined metabolism of Medicago truncatula and its nitrogen fixing symbiont Sinorhizobium meliloti elucidated by genome-scale metabolic models	bioRxiv
**Weber,T. and Kim,H.U.**	2016	The secondary metabolite bioinformatics portal: Computational tools to facilitate synthetic biology of secondary metabolite production	Synthetic and Systems Biotechnology
**Kim,W.J., Kim,H.U. and Lee,S.Y.**	2017	Current state and applications of microbial genome-scale metabolic models	Current Opinion in Systems Biology
**Hädicke,O. and Klamt,S.**	2017	EColiCore2: a reference network model of the central metabolism of Escherichia coli and relationships to its genome-scale parent model	Scientific Reports
**Lopes,H. and Rocha,I.**	2017	Genome-scale modeling of yeast: chronology, applications and critical perspectives	FEMS Yeast Research
**102. Thor,S., Peterson,J.R. and Luthey-Schulten,Z.**	2017	Genome-Scale Metabolic Modeling of Archaea Lends Insight into Diversity of Metabolic Function	Archaea
**Chiappino-Pepe,A., Pandey,V., Ataman,M. and Hatzimanikatis,V.**	2017	Integration of metabolic, regulatory and signaling networks towards analysis of perturbation and dynamic responses	Current Opinion in Systems Biology
**Mendes-Soares,H. and Chia,N.**	2017	Community metabolic modeling approaches to understanding the gut microbiome: Bridging biochemistry and ecology	Free Radical Biology and Medicine
**Fondi,M. and Fani,R.**	2017	Constraint-based metabolic modelling of marine microbes and communities	Marine Genomics
**Karlsen,E., Schulz,C. and Almaas,E.**	2018	Automated generation of genome-scale metabolic draft reconstructions based on KEGG	BMC Bioinformatics
**Lakshmanan,M., Koduru,L. and Lee,D.-Y.**	2018	Software Applications for Phenotype Analysis and Strain Design of Cellular Systems	In Emerging Areas in Bioengineering

**100. Muller,E.E.L., Faust,K., Widder,S., Herold,M., Martínez Arbas,S. and Wilmes,P.**	2018	Using metabolic networks to resolve ecological properties of microbiomes	Current Opinion in Systems Biology
**Rau,M.H. and Zeidan,A.A.**	2018	Constraint-based modeling in microbial food biotechnology	Biochemical Society Transactions
**Machado,D., Andrejev,S., Tramontano,M. and Patil,K.R.**	2018	Fast automated reconstruction of genome-scale metabolic models for microbial species and communities	Nucleic Acids Research
**Choi,K.R., Jang,W.D., Yang,D., Cho,J.S., Park,D. and Lee,S.Y.**	2019	Systems Metabolic Engineering Strategies: Integrating Systems and Synthetic Biology with Metabolic Engineering	Trends in Biotechnology
**101. Angione,C.**	2019	Human Systems Biology and Metabolic Modelling: A Review—From Disease Metabolism to Precision Medicine	BioMed Research International
**Castillo,S., Patil,K.R. and Jouhten,P.**	2019	Yeast Genome-Scale Metabolic Models for Simulating Genotype–Phenotype Relations	Yeasts in Biotechnology and Human Health
**Gu,C., Kim,G.B., Kim,W.J., Kim,H.U. and Lee,S.Y.**	2019	Current status and applications of genome-scale metabolic models	Genome Biology 2019 20:1
**Santibáñez,R., Garrido,D. and Martin,A.J.M.**	2020	Atlas : automatic modeling of regulation of bacterial gene expression and metabolism using rule-based languages	Bioinformatics
**Alballa,M., Aplop,F. and Butler,G.**	2020	TranCEP: Predicting the substrate class of transmembrane transport proteins using compositional, evolutionary, and positional information	PLOS ONE
**Lam,T.J., Stamboulian,M., Han,W. and Ye,Y.**	2020	Model-based and phylogenetically adjusted quantification of metabolic interaction between microbial species	PLOS Computational Biology
**Simensen,V., Voigt,A. and Almaas,E.**	2020	High-quality genome-scale metabolic model of Aurantiochytrium sp. T66	bioRxiv
**Little,B.J., Blackwood,D.J., Hinks,J., Lauro,F.M., Marsili,E., Okamoto,A., Rice,S.A., Wade,S.A. and Flemming,H.-C.**	2020	Microbially influenced corrosion—Any progress?	Corrosion Science
**Xu,X., Liu,Y., Du,G. and Liu,L.**	2020	Systems biology, synthetic biology, and metabolic engineering	In Systems and Synthetic Metabolic Engineering
**Seif,Y. and Palsson,B.Ø.**	2021	Path to improving the life cycle and quality of genome-scale models of metabolism	Cell Systems

**Esvap,E. and Ulgen,K.O.**	2021	Advances in Genome-Scale Metabolic Modeling toward Microbial Community Analysis of the Human Microbiome	ACS Synthetic Biology
**Kuriya,Y., Inoue,M., Yamamoto,M., Murata,M. and Araki,M.**	2021	Knowledge extraction from literature and enzyme sequences complements FBA analysis in metabolic engineering	Biotechnology Journal
**Moscardó García,M., Pacheco,M., Bintener,T., Presta,L. and Sauter,T.**	2021	Importance of the biomass formulation for cancer metabolic modeling and drug prediction	iScience
**Saraiva,J.P., Worrich,A., Karakoç,C., Kallies,R., Chatzinotas,A., Centler,F. and Nunes da Rocha,U.**	2021	Mining Synergistic Microbial Interactions: A Roadmap on How to Integrate Multi-Omics Data	Microorganisms
**Chung,W.Y., Zhu,Y., Mahamad Maifiah,M.H., Shivashekaregowda,N.K.H., Wong,E.H. and Abdul Rahim,N.**	2021	Novel antimicrobial development using genome-scale metabolic model of Gram-negative pathogens: a review	The Journal of Antibiotics
**Patra,P., Das,M., Kundu,P. and Ghosh,A.**	2021	Recent advances in systems and synthetic biology approaches for developing novel cell-factories in non-conventional yeasts	Biotechnology Advances
**Sangha,G.S., Goergen,C.J., Prior,S.J., Ranadive,S.M. and Clyne,A.M.**	2021	Preclinical techniques to investigate exercise training in vascular pathophysiology	American Journal of Physiology-Heart and Circulatory Physiology
**Frades,I., Foguet,C., Cascante,M. and Araúzo-Bravo,M.J.**	2021	Genome Scale Modeling to Study the Metabolic Competition between Cells in the Tumor Microenvironment	Cancers
**Wendering,P. and Nikoloski,Z.**	2022	COMMIT: Consideration of metabolite leakage and community composition improves microbial community reconstructions	PLOS Computational Biology
**Camborda,S., Weder,J.-N. and Töpfer,N.**	2022	CobraMod: a pathway-centric curation tool for constraint-based metabolic models	Bioinformatics
**Chu,L., Li,S., Dong,Z., Zhang,Y., Jin,P., Ye,L., Wang,X. and Xiang,W.**	2022	Mining and engineering exporters for titer improvement of macrolide biopesticides in Streptomyces	Microbial Biotechnology

**Carey,M.A., Medlock,G.L., Stolarczyk,M., Petri,W.A., Guler,J.L. and Papin,J.A.**	2022	Comparative analyses of parasites with a comprehensive database of genome-scale metabolic models	PLOS Computational Biology
**Pathania,R., Srivastava,A., Srivastava,S. and Shukla,P.**	2022	Metabolic systems biology and multi-omics of cyanobacteria: Perspectives and future directions	Bioresource Technology
**Saa,P., Urrutia,A., Silva-Andrade,C., Martín,A.J. and Garrido,D.**	2022	Modeling approaches for probing cross-feeding interactions in the human gut microbiome	Computational and Structural Biotechnology Journal
**Navid,A.**	2022	Curating COBRA Models of Microbial Metabolism	Microbial Systems Biology

References

1. Kanehisa, M., Furumichi, M., Tanabe, M., Sato, Y., Morishima, K.: (2017) KEGG: New perspectives on genomes, pathways, diseases and drugs. *Nucleic Acids Res*.

2. Sayers,E.W., Beck,J., Bolton,E.E., Bourexis,D., Brister,J.R., Canese,K., Comeau,D.C., Funk,K., Kim,S., Klimke,W., *et al.* (2021) Database resources of the National Center for Biotechnology Information. *Nucleic Acids Res.*, **49**, D10–D17

3. Seaver,S.M.D., Liu,F., Zhang,Q., Jeffryes,J., Faria,J.P., Edirisinghe,J.N., Mundy,M., Chia,N., Noor,E., Beber,M.E., *et al.* (2021) The ModelSEED Biochemistry Database for the integration of metabolic annotations and the reconstruction, comparison and analysis of metabolic models for plants, fungi and microbes. *Nucleic Acids Res.*, **49**, D575–D588.

4. Saier,M.H., Reddy,V.S., Tsu,B. V., Ahmed,M.S., Li,C. and Moreno-Hagelsieb,G. (2016) The Transporter Classification Database (TCDB): recent advances. *Nucleic Acids Res.*, **44**, D372–D379.

5. The UniProt Consortium (2019). UniProt: a worldwide hub of protein knowledge *Nucleic Acids Res.*, **47**, D506–D515.

6. Cruz,F., Lagoa,D., Mendes,J., Rocha,I., Ferreira,E.C., Rocha,M. and Dias,O. (2019) SamPler – a novel method for selecting parameters for gene functional annotation routines. *BMC Bioinformatics*, **20**, 454.

7. Lagoa,D., Faria,J.P., Liu,F., Cunha,E., Ferreira,E.C., Henry,C.S. and Dias,O. (2021) TranSyT: the Transport Systems Tracker.

8. Yu,N.Y., Wagner,J.R., Laird,M.R., Melli,G., Rey,S., Lo,R., Dao,P., Sahinalp,S.C., Ester,M., Foster,L.J., *et al.* (2010) PSORTb 3.0: improved protein subcellular localization prediction with refined localization subcategories and predictive capabilities for all prokaryotes. *Bioinformatics*, **26**, 1608–1615.

9. Horton,P., Park,K.-J., Obayashi,T., Fujita,N., Harada,H., Adams-Collier,C.J. and Nakai,K. (2007) WoLF PSORT: protein localization predictor. *Nucleic Acids Res.*, **35**, W585–W587.

10. Goldberg,T., Hecht,M., Hamp,T., Karl,T., Yachdav,G., Ahmed,N., Altermann,U., Angerer,P., Ansorge,S., Balasz,K., *et al.* (2014) LocTree3 prediction of localization. *Nucleic Acids Res.*, **42**, W350–W355.

11. Dias,O., Liu,F., Rocha,M., Ferreira,E.C. and Rocha,I. (2014) Prediction of protein subunits using KEGG BRITE.

12. Santos,S. and Rocha,I. (2016) Estimation of biomass composition from genomic and transcriptomic information. *J. Integr. Bioinform.*, **13**, 1–14.

13. Oliveira,A., Cunha,E., Cruz,F., Capela,J., Sequeira,J., Sampaio,M. and Dias,O. (2021) Towards a Multivariate Analysis of Genome-Scale Metabolic Models Derived from the BiGG Models Database. *Lecture Notes in Networks and Systems*, **325 LNNS**, 136–144.

14. Cruz,F., Capela,J., Ferreira,E.C., Rocha,M. and Dias,O. (2021) BioISO: an objective-oriented application for assisting the curation of genome-scale metabolic models.

15. Lieven,C., Beber,M.E., Olivier,B.G., Bergmann,F.T., Ataman,M., Babaei,P., Bartell,J.A., Blank,L.M., Chauhan,S., Correia,K., *et al.* (2020) MEMOTE for standardized genome-scale metabolic model testing. *Nat. Biotechnol.*, **38**, 272–276.

16. King,Z.A., Dräger,A., Ebrahim,A., Sonnenschein,N., Lewis,N.E. and Palsson,B.O. (2015) Escher: A Web Application for Building, Sharing, and Embedding Data-Rich Visualizations of Biological Pathways. *PLOS Comput. Biol.*, **11**, e1004321.

17. Chazalviel,M., Frainay,C., Poupin,N., Vinson,F., Merlet,B., Gloaguen,Y., Cottret,L. and Jourdan,F. (2018) MetExploreViz: web component for interactive metabolic network visualization. *Bioinformatics*, **34**, 312–313.

18. Resende,T., Correia,D.M., Rocha,M. and Rocha,I. (2013) Re-annotation of the genome sequence of Helicobacter pylori 26695. *J. Integr. Bioinform.*, **10**, 233.

19. Dias,O., Saraiva,J., Faria,C., Ramirez,M., Pinto,F. and Rocha,I. (2019) iDS372, a Phenotypically Reconciled Model for the Metabolism of Streptococcus pneumoniae Strain R6. *Front. Microbiol.*, **10**.

20. Cunha,E., Cruz,F., Silva,M., Ribeiro,C., Rocha,I., Zeidan,A. and Dias,O. (2021) Unveiling metabolic traits of dairy bacteria through in silico genome-scale metabolic modelling.

21. Pereira,B., Miguel,J., Vilaça,P., Soares,S., Rocha,I. and Carneiro,S. (2018) Reconstruction of a genome-scale metabolic model for Actinobacillus succinogenes 130Z. *BMC Syst. Biol.*, **12**, 61.

22. Portela,C., Villas-Bôas,S., Rocha,I. and Ferreira,E.C. (2013) Genome scale metabolic network reconstruction of the pathogen Enterococcus faecalis. *IFAC Proc. Vol.*, **46**, 131–136.

23. Dias,O., Pereira,R., Gombert,A.K., Ferreira,E.C. and Rocha,I. (2014) iOD907, the first genome-scale metabolic model for the milk yeast Kluyveromyces lactis. *Biotechnol. J.*, **9**, 776–790.

24. Viana,R., Dias,O., Lagoa,D., Galocha,M., Rocha,I. and Teixeira,M.C. (2020) Genome-Scale Metabolic Model of the Human Pathogen Candida albicans: A Promising Platform for Drug Target Prediction. *J. Fungi*, **6**, 171.

25. Cunha,E., Silva,M., Chaves,I., Demirci,H., Lagoa,D., Lima,D., Rocha,M., Rocha,I. and Dias,O. iEC7871 Quercus suber model: the first multi-tissue diel cycle genome-scale metabolic model of a woody tree. https://doi.org/10.1101/2021.03.09.434537.
