# Supplementary material for: merlin, an improved framework for the reconstruction of high-quality genome-scale metabolic models
Source: Nucleic Acids Res. 2022 Jun 13;50(11):6052–66. doi: 10.1093/nar/gkac459 (PMC9226533; doi:10.1093/nar/gkac459)
Supplement: gkac459_Supplemental_Files [file gkac459_supplemental_files.zip › S2.pdf]

merlin v4.0: an updated platform for the reconstruction of high-quality genome-scale metabolic models

João Capela[1,2], Davide Lagoa[1,2], Ruben Rodrigues[1,2], Emanuel Cunha[1,2], Fernando Cruz[1,2] Ana Barbosa[1,2], José Bastos[1,2], Diogo Lima[1,2], Eugénio C. Ferreira[1,2], Miguel Rocha[1,2], Oscar Dias[1,2,*]

[1] Centre of Biological Engineering, University of Minho, 4710-057, Braga, Portugal

[2] LABBELS – Associate Laboratory, Braga/Guimarães, Portugal

* To whom correspondence should be addressed.

Tel: 00351 253 604 422

Email: odias@deb.uminho.pt

Supplementary data 2 – draft models' assessment

Drafts Assessment approach

These draft reconstructions were compared with manually curated and validated models—the GSMMs of *L. plantarum, B. pertussis,* and *T. gondii* were iLP728, iBP1870, and ToxoNet1, respectively.

iBP1870 contains 770 genes and 1672 reactions. Regarding the latter, 564 (33.73%) are sink, demand, exchange or transport reactions. Out of the remaining 1108, only 933 were converted to other database identifiers.

iLP728 contains 771 reactions and 728 genes. Regarding the latter, 228 (29,57%) were either sink, demand, exchange or transport reactions. Out of the remaining 543, 479 were converted to other database identifiers.

ToxoNet1 is composed of 4414 reactions and 940 genes. Regarding the latter, 3316 (75,12%) were either sink, demand, exchange or transport reactions. Out of the remaining 1098, 957 were converted to other database identifiers.

Table S1 – Models' features

	Total reactions	Sinks	Exchange	Transport	Demand	Compartments	Genes
iBP1870	1672	0	204	355	5	3	770
iLP728	771	0	113	216	1	2	728
ToxoNet1	4414	0	2056	1251	0	4	940

Evaluated Tools

AuReMe

AuReMe (Automatic Reconstruction of Metabolic Models) is a Docker-based software that allows reconstructing simulation-ready GSMMs using others as templates or inheriting a genome annotation from Pathway Tools.

The AuReME Docker image version 2.4 was utilised to generate the draft models. AuReMe requires other models as templates and the genome files of the organisms under study, including the template ones. In this sense, the genome FASTA files were preprocessed to contain only the locus tag in the header of each gene. Moreover, the template models were imported to *merlin* in SBML level 3 and exported in SBML level 2 format. Otherwise, AuReMe could not be run as it does not support SBML level 3 template models. Then, an orthology-based reconstruction was generated using the Ortho-Finder algorithm.

PathwayTools

Pathway Tools is software designed for the development of organism-specific metabolic databases. The application is developed in Lisp and provides a graphical interface that allows the user to visualise the whole constructed network. Moreover, it includes the assisted analysis of experimental data in the metabolic network.

The Pathway Tools version 25.5 was downloaded and utilised to generate draft models of the mentioned organisms under study. For this matter, we uploaded the genome file in GenBank format and performed the metabolic network reconstruction with the Pathologic algorithm using default parameters. Moreover, transport reactions were generated and included. Finally, the draft model was exported in an SBML format.

CarveMe

CarveMe is a python package focused on fast reconstructing microbial metabolic models. It provides a top-down approach that creates simulation-ready models based on BiGG universal database. This tool uses Diamond to prioritise reactions with stronger genetic evidence (by similarity).

The protein sequences were used as input in FASTA format using default parameters, providing simulation-ready GSM models in SBML format. The gap-filling mode of this tool was not used.

RAVEN

RAVEN (Reconstruction, Analysis and Visualization of Metabolic Networks) is a command-line tool compliant with COBRA Toolbox v3, thus running in MATLAB. It provides functionalities to reconstruct models from scratch or use template models, merge networks from different databases and curate from the command line.

The protein FASTA files were used as input to version 2.5.3 of the tool, using the KEGG's "prok90_kegg94" and "euk90_kegg94" templates for prokaryotic and eukaryotic organisms, respectively. The tool was used with default parameters, except for the "keepUndefinedStoich" and "keepIncomplete" (were set as False), and the cutOff value (adjusted to 10^{-30}).

ModelSEED

ModelSEED is a web tool to analyse and reconstruct GSMMs. The genome annotation is performed by RAST within ModelSEED, reconstructing the metabolic network based on that annotation. Moreover, it provides a graphical interface to analyse and gap-fill the model and run simulations.

ModelSEED version 2 (November 2021) was used to reconstruct the draft models of the organisms under study. The genome FASTA files were inputted directly through the web interface, and the template models were defined by each type of organism (gram-positive bacteria for *L. plantarum* and gram-negative bacteria for *B. pertussis*). Finally, we selected a complete medium for the reconstruction.

AutoKEGGRec

AutoKEGGRec v1.01 is a command-line tool that runs in MATLAB and provides compliance with COBRA Toolbox v3. This tool is highly suitable to generate multiple GSMMs only in one run and can also generate metabolic models for communities. The reconstruction is dependent on the availability of data for the target organism in KEGG.

The KEGG's organism identifiers for *L. plantarum*, *B. pertussis*, and *T. gondii* were used as input, using the "SingleRecs" mode, suitable for reconstructing GSM models of individual organisms. After running the tool, the models were exported in SBML format.

merlin

Regarding *merlin,* we generated several more models to validate all the new and updated features. In this sense, the first step encompassed uploading the genome FASTA file, which was aligned against the Swiss-Prot and TrEBML databases, sequentially, using BLAST or Diamond. The default BLAST and Diamond parameters were inputted; however, two Diamond sensitivity modes were assessed: "fast" and "sensitive". Afterwards, the automatic annotation and *SamPler* were used to annotate the genomes based on the BLAST/Diamond results. The species and genus selected for *automatic annotation* are shown in Tables S1, S2, and S3. The selection of organisms was based on phylogenetic trees obtained by applying a neighbour-joining method to a multiple alignments generated by Clustal Omega of ribosomal RNA (16S for prokaryotes and 18S for eukaryotes) of species well-represented at

Swiss-Prot. The results of this selection are described in Supplementary material 3. Furthermore, transport reactions were generated with *TranSyT*. Finally, GPR rules were constructed using blast+ version 2.11.0.

Table S2 – Automatic workflow's ranking for *B. pertussis*.

**genus/species**	**organism**	**e-value threshold**	**reviewed**
species	*Bordetella pertussis*	1.0E-30	true
genus	*Bordetella*	1.0E-30	true
species	*Pseudomonas putida*	1.0E-30	true
species	*Escherichia coli*	1.0E-30	true
species	*Klebsiella pneumoniae*	1.0E-30	true
species	any	1.0E-30	true
species	*Bordetella pertussis*	1.0E-30	false
genus	*Bordetella*	1.0E-30	false
species	*Pseudomonas putida*	1.0E-30	false

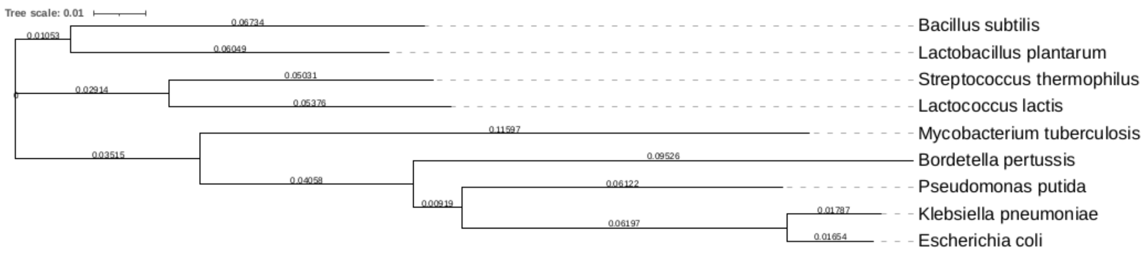

Figure S1 – Phylogenetic tree obtained by applying a neighbour-joining method to a multiple alignment generated by Clustal Omega of 16S ribosomal RNA.

Table S3 – Automatic workflow's ranking for *L. plantarum*.

**genus/species**	**organism**	**e-value threshold**	**reviewed**
species	*Lactobacillus plantarum* (strain ATCC BAA-793 / NCIMB 8826 / WCFS1)	1.0E-30	true
genus	*Lactobacillus*	1.0E-30	true
species	*Bacillus subtilis*	1.0E-30	true
species	*Streptococcus thermophilus*	1.0E-30	true
species	*Lactococcus lactis*	1.0E-30	true
species	any	1.0E-30	true
species	*Lactobacillus plantarum (strain ATCC BAA-793 / NCIMB 8826 / WCFS1)*	1.0E-30	false
genus	*Lactobacillus*	1.0E-30	false
species	*Bacillus subtilis*	1.0E-30	false

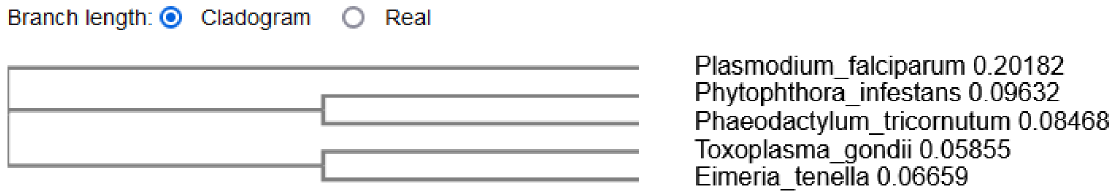

Figure S2 – Phylogenetic tree obtained by applying a neighbour-joining method to a multiple alignment generated by Clustal Omega of 18S ribosomal RNA.

Table S4 – Automatic workflow's ranking for *T. gondii*.

**genus/species**	**organism**	**e-value threshold**	**reviewed**
species	*Toxoplasma gondii* ME49	1.0E-30	true
genus	*Toxoplasma*	1.0E-30	true
species	*Eimeria tenella*	1.0E-30	true
species	*Phaeodactylum tricornutum*	1.0E-30	true
species	*Plasmodium falciparum*	1.0E-30	true
species	any	1.0E-30	true
species	*Toxoplasma gondii* ME49	1.0E-30	false
genus	*Toxoplasma*	1.0E-30	false
species	*Plasmodium falciparum*	1.0E-30	false

merlin-BIT models were generated on the "use specific BiGG models information" option, where we selected the template models.

merlin models will be referred to hereafter using the following nomenclature:

- *merlin*-DFA – *merlin* model using Diamond "fast" mode and Automatic workflow;
- *merlin*-DFS – *merlin* model using Diamond "fast" mode and *SamPler*;
- *merlin*-DSA – *merlin* model using Diamond "sensitive" mode and Automatic workflow;
- *merlin*-DSS – *merlin* model using Diamond "sensitive" mode and *SamPler*;
- *merlin*-BA – *merlin* model using BLAST and Automatic workflow;
- *merlin*-BS – *merlin* model using BLAST and *SamPler*.

Results of models' assessment

Bordetella pertussis

Reactions

Seemingly, the best draft model obtained with *merlin* was generated with BIT. It achieved top performance on the most critical metrics: F1, Recall and JD. However, *merlin-DSA* and *merlin-DSS* obtained the best Precision and Ratio among *merlin* models. Besides *merlin-BIT* results, *merlin-BS* obtained the best F1 and JD among the non-template-based models. Nevertheless, the other *merlin* draft models achieved similar metric values, as the difference was on the order of the centesimal at most.

Table S5 – Reactions results for all the models of *B. pertussis*.

model	Reactions number	Removed reactions	Converted reactions	Non-converted reactions	Duplicated reactions	recall	precision	f1	TP	FP	FN	ratio	jaccard distance
***merlin*-DFS**	1891	601	607	683	6	0,31	0,47	0,37	282	319	642	0,88	0,77
***merlin*-DFA**	1891	601	607	683	6	0,31	0,47	0,37	282	319	642	0,88	0,77
***merlin*-DSA**	1761	570	560	631	6	0,29	0,49	0,37	272	282	652	0,96	0,77
***merlin*-DSS**	1761	570	560	631	6	0,29	0,49	0,37	272	282	652	0,96	0,77
***merlin*-BS**	1813	560	591	662	6	0,31	0,48	0,37	282	303	642	0,93	0,77
**merlin-BA**	1819	562	589	668	6	0,30	0,48	0,37	279	304	645	0,92	0,77
***merlin*-BIT**	1526	337	1157	32	15	0,51	0,41	0,46	468	674	445	0,69	0,71
**AuReMe**	881	178	702	1	10	0,43	0,57	0,49	396	296	520	1,34	0,67
**autoKEGGrec**	884	0	575	309	7	0,34	0,55	0,42	310	258	614	1,20	0,74
**CarveMe**	2307	924	1375	8	63	0,53	0,37	0,43	482	830	426	0,58	0,72
**ModelSEED**	1336	185	554	597	1	0,29	0,49	0,37	273	280	660	0,98	0,77
**PathwayTools**	1334	35	379	920	1	0,25	0,62	0,36	235	143	695	1,64	0,78
**RAVEN**	3577	0	1133	2444	9	0,37	0,30	0,33	340	784	585	0,43	0,80

Genes

merlin's models performed similarly for gene sets, with no significant differences. However, it is worth noting that using *SamPler* seems to improve the annotation slightly, even if the sample annotation is performed automatically. Overall, the models generated with *merlin* outperformed almost all other models except for CarveMe in the most critical metrics.

Table S6 – Genes results for all the models of *B. pertussis*.

model	Genes number	recall	precision	f1	TP	FP	FN	ratio	jaccard distance
***merlin*-DFS**	832	0,66	0,61	0,63	507	325	263	1,56	0,54
***merlin*-DFA**	829	0,66	0,61	0,63	507	322	263	1,57	0,54
***merlin*-DSA**	738	0,63	0,66	0,65	487	251	283	1,94	0,52
***merlin*-DSS**	737	0,63	0,66	0,65	487	250	283	1,95	0,52
***merlin*-BS**	861	0,68	0,61	0,64	521	340	249	1,53	0,53
**merlin-BA**	858	0,67	0,60	0,64	519	339	251	1,53	0,53
***merlin*-BIT**	814	0,65	0,61	0,63	497	317	273	1,57	0,54
**AuReMe**	452	0,47	0,80	0,59	362	90	408	4,02	0,58
**autoKEGGrec**	934	0,68	0,56	0,61	520	414	250	1,26	0,56
**CarveMe**	1026	0,76	0,57	0,65	584	442	186	1,32	0,52
**ModelSEED**	675	0,57	0,65	0,61	441	234	329	1,88	0,56
**PathwayTools**	780	0,60	0,60	0,60	465	315	305	1,48	0,57
**RAVEN**	1415	0,75	0,41	0,53	576	839	194	0,69	0,64

Lactobacilus plantarum

Reactions

merlin-BIT outperformed the other models in all metrics except AuReMe and CarveMe (only in Recall). As for the other merlin models, *merlin-BS* was slightly better than *merlin-BA* for all metrics except Recall, while the other models surpassed *merlin-DFA*. Furthermore, it is worth noting that three out of the five top-performance models regarding F1, Ratio and JD were generated with *merlin*.

Table S7 – Reactions results for all the models of *L. plantarum*.

model	Reactions number	Removed reactions	Converted reactions	Non-converted reactions	Duplicated reactions	recall	precision	f1	TP	FP	FN	ratio	jaccard distance
***merlin*-DFS**	1201	320	427	454	6	0,43	0,49	0,46	205	216	274	0,95	0,71
***merlin*-DFA**	1193	320	414	459	6	0,42	0,49	0,45	201	207	278	0,97	0,71
***merlin*-DSA**	1205	324	432	449	6	0,43	0,48	0,46	206	220	273	0,94	0,71
***merlin*-DSS**	1234	326	436	472	4	0,45	0,50	0,47	214	218	265	0,98	0,69
***merlin*-BS**	1251	324	461	466	5	0,49	0,52	0,51	237	219	242	1,08	0,66
**merlin-BA**	1307	334	478	495	5	0,50	0,51	0,50	240	233	239	1,03	0,66
***merlin*-BIT**	665	105	536	24	0	0,63	0,56	0,60	302	234	177	1,29	0,58
**AuReMe**	423	47	367	9	0	0,55	0,71	0,62	262	105	217	2,50	0,55
**autoKEGGrec**	673	0	441	232	7	0,49	0,54	0,51	235	199	244	1,18	0,65
**CarveMe**	1453	537	913	3	66	0,70	0,39	0,50	333	514	146	0,65	0,66
**ModelSEED**	1274	238	509	527	1	0,47	0,44	0,46	226	282	253	0,80	0,70
**PathwayTools**	2077	810	365	902	1	0,41	0,54	0,46	195	169	284	1,15	0,70
**RAVEN**	3184	0	1093	2091	9	0,58	0,26	0,36	278	806	201	0,34	0,78

Genes

merlin-DFA and *merlin-DFS* performed worse than the other drafts generated with *merlin*. These results suggest that Diamond's alignment results using the "fast" mode can affect the draft model construction while using the "sensitive" mode could not significantly improve or impair the reconstruction compared with BLAST. Furthermore, *merlin-BS* surpassed the other *merlin* models whose genome annotation was only performed with the automatic annotation algorithm (*merlin-BA, merlin-DFA*, and *merlin-DSA*), suggesting that *SamPler* can improve the similarity between a draft and a curated model.

Table S8 – Genes results for all the models of *L. plantarum*.

model	Genes number	recall	precision	f1	TP	FP	FN	ratio	jaccard distance
***merlin*-DFS**	606	0,61	0,73	0,67	444	162	284	2,74	0,50
***merlin*-DFA**	595	0,60	0,73	0,66	435	160	293	2,72	0,51
***merlin*-DSA**	635	0,64	0,73	0,68	464	171	264	2,71	0,48
***merlin*-DSS**	631	0,63	0,72	0,67	456	175	272	2,61	0,50
***merlin*-BS**	675	0,67	0,72	0,70	488	187	240	2,61	0,47
**merlin-BA**	725	0,67	0,68	0,68	491	234	237	2,10	0,49
***merlin*-BIT**	552	0,61	0,81	0,70	445	107	283	4,16	0,47
**AuReMe**	317	0,40	0,92	0,56	291	26	437	11,19	0,61
**autoKEGGrec**	807	0,73	0,66	0,69	531	276	197	1,92	0,47
**CarveMe**	808	0,67	0,60	0,63	485	323	243	1,50	0,54
**ModelSEED**	718	0,67	0,68	0,67	488	230	240	2,12	0,49
**PathwayTools**	856	0,70	0,59	0,64	507	349	221	1,45	0,53
**RAVEN**	1295	0,84	0,47	0,60	609	686	119	0,89	0,57

Toxoplasma gondii

Reactions

merlin-DFS, -DSA, and *merlin-DSS* models presented neglectable differences for all metrics, which indicate they are extremely similar. On the other hand, *merlin-BIT* obtained the worst results overall except for Precision and Ratio.

Table S9 – Reactions results for all the models of *T. gondii*.

model	Reactions number	Removed reactions	Converted reactions	Non-converted reactions	Duplicated reactions	recall	precision	f1	TP	FP	FN	ratio	jaccard distance
***merlin*-DFS**	1345	215	1125	5	8	0,61	0,51	0,55	570	547	370	1,04	0,62
***merlin*-DFA**	1446	228	1211	7	8	0,63	0,49	0,55	593	610	347	0,97	0,62
***merlin*-DSA**	1335	210	1120	5	8	0,60	0,51	0,55	567	545	373	1,04	0,62
***merlin*-DSS**	1335	210	1120	5	8	0,60	0,51	0,55	567	545	373	1,04	0,62
***merlin*-BS**	1441	228	1206	7	7	0,63	0,49	0,55	591	608	349	0,97	0,62
**merlin-BA**	1441	228	1206	7	7	0,63	0,49	0,55	591	608	349	0,97	0,62
***merlin*-BIT**	564	106	134	324	9	0,09	0,71	0,17	89	36	853	2,47	0,91
**AuReMe**	432	89	209	134	7	0,18	0,83	0,29	167	35	774	4,77	0,83
**autoKEGGrec**	567	0	564	3	7	0,43	0,72	0,53	400	157	541	2,55	0,64
**PathwayTools**	1533	243	356	934	2	0,26	0,69	0,38	245	109	695	2,25	0,77
**RAVEN**	2655	0	2640	15	14	0,90	0,32	0,48	847	1779	93	0,48	0,69

Genes

Apart from *merlin-DSA* and *-DSS* outperforming the other merlin models, their differences were negligible. Moreover, using *SamPler* was not as relevant as for the prokaryote reconstructions.

Table S10 – Genes results for all the models of *T. gondii*.

model	Genes number	recall	precision	f1	TP	FP	FN	ratio	jaccard distance
***merlin*-DFS**	591	0,60	0,53	0,56	315	276	211	1,14	0,61
***merlin*-DFA**	715	0,65	0,47	0,54	333	382	181	0,87	0,63
***merlin*-DSA**	589	0,60	0,53	0,57	315	274	210	1,15	0,61
***merlin*-DSS**	589	0,60	0,53	0,57	315	274	210	1,15	0,61
***merlin*-BS**	708	0,63	0,46	0,53	324	384	193	0,84	0,64
**merlin-BA**	708	0,63	0,46	0,53	324	384	193	0,84	0,64
***merlin*-BIT**	104	0,07	0,62	0,13	64	40	794	1,60	0,93
**AuReMe**	243	0,20	0,62	0,31	150	93	587	1,61	0,82
**autoKEGGrec**	772	0,51	0,38	0,43	293	479	287	0,61	0,72
**PathwayTools**	1236	0,72	0,28	0,40	345	891	133	0,39	0,75
**RAVEN**	1017	0,93	0,38	0,54	388	629	30	0,62	0,63
